# Report of the Committee Appointed to Memorialize Congress in Regard to the Medical Department of the Army

**Published:** 1866-03

**Authors:** Charles S. Tripler, Chris. C. Cox

**Affiliations:** Cincinnati, Ohio; Cincinnati, Ohio


### REPORT OF THE COMMITTEE APPOINTED TO MEMORIALIZE CONGRESS IN REGARD TO THE MEDICAL DEPARTMENT OF THE ARMY.

CINCINNATI, OHIO, May 9, 1865.

The undersigned of the Committee appointed by the American Medical Association to memoralize Congress in regard to the passage of a law for the Medical Department of the Army, and to present the draft of such a law to each Senator and Representative, have the honor to report:—

That they took the subject immediately into consideration, but that the time they could command was too limited to enable them to prepare a memorial and draft a law before the adjournment of the session of 1864 and 1865. After an interchange of views among the members of the Committee, necessarily conducted through the mails, a memorial and draft of a law were agreed upon by the majority of the Committee, printed and distributed as directed by the Association, at the commencement of the last session of Congress. This memorial and draft of a law were in the words following, to wit:—

*To the Senate and House of Representatives of the United States:*

The undersigned, a Committee appointed by the American Medical Association, at a meeting held in the city of New York, in June, 1864, to memoralize your Honorable Bodies in regard to the passage of a law for the better organization of the Medical Department of the Army of the United States, in the discharge of the duty imposed upon them, respectfully represent—

That if the experience of the previous wars in which the country has been engaged had not been sufficient to indicate the importance and necessity of clothing the medical officers of the army with an absolute military rank, that should settle their authority to execute their own functions unquestioned by officers of other branches of the service, the magnitude of the present contest, and its all-pervading interests, have, in their opinion, forever determined this fact. The necessities of present circumstances have forced upon medical officers the exercise of military functions and command, without and even against the authority of law, in some instances, but which is absolutely indispensable to the existence of a Hospital Department. The Act of February 11, 1847, which first conferred military rank upon medical officers, expressly forbids them to exercise military command in the line, or another staff department. Still from necessity, and in obedience to the orders of the Secretary of War, they now exercise such command, and that under General Orders No. 212, July 9, 1863, emanating from the War Department.

Surgeons in charge of general hospitals are and must be the commanding officers thereof. They are responsible as well for the discipline and administration of those establishments as for the medical and surgical treatment of the sick and wounded. From the nature of the case, none other than a medical officer can discharge these strictly military duties, and wherever an attempt has been made to divorce the military and professional functions in a general hospital, the result has been conflict of opinion and authority, discontent, altercation, and paralyzed efficiency.

Were the claim to an effective and indisputable rank for the Medical Department novel in principle in our service, it might be plausibly controverted; but when the propriety and advantage of such rank in staff departments have been so completely settled, as they have been by the examples of the Quartermaster's and Subsistence Departments, it cannot now be successfully refuted.

Brought into immediate contact with, and exercising the immediate control over thousands of soldiers, charged with the care of their pay, subsistence, clothing, and discipline while in the General Hospitals, the Medical Department alone is assimilated in functions to the officers of the line, and is *de facto* compelled to exercise military command over the persons of soldiers, and whatever of failure there may have been to meet the full requirements of their duties in these respects, is, in our opinion, distinctly to be traced to the defenceless position in which they are left in regard to the interference of other officers possessing the immense advantage of unqualified military rank, with their own management of their own departments.

The grade of the medical officer in command of a general hospital should, in our judgment, never be less than that of a field-officer. Many of these establishments contain more than one thousand soldiers, some of them more than three thousand, a command equal to that of a brigadier-general. The medical directors of armies and military departments have only the rank of majors, and even this restricted, while the chief quartermasters of the same are invested with the unrestricted rank of colonels—the latter exercising no command over soldiers, while the former have from ten to twenty thousand military persons under their control. They are at the same time responsible for the careful and economical distribution of vast amounts of public property, and without the experience and ability in this special department, which long and careful training alone can give, the money value of the loss of life which would result would certainly equal the expenditures of the Quartermaster's Department, vast as they are. With at least equal responsibilities, and equal zeal, and equal devotion, the medical officer is denied an equal rank with the quartermaster, and even the limited rank which is conceded to him, is so clogged with restrictions as to be of little avail for the public good.

We, therefore, pray your Honorable Bodies to give these evils your serious consideration, persuaded that the result will be such a remedy as will satisfy the claims of equity, of justice, and of the best interests of the service.

The Committee having been further instructed to prepare a bill for the consideration of Congress, and to "embrace in its provisions a further separation of the Medical Department from the Commanding Officers of the Line, in order to have a more perfect and unrestrained control of its interests and greater efficiency in that branch of the service, in the fulfilment of this duty, beg leave to present the following sketch of such a bill as they think would meet the views of the American Medical Association, and secure the objects they desire:

1. The Medical Department of the Regular Army shall consist of—

One Surgeon-General—Brigadier-General:—

| | |
|---|---|
| One Assist.-Surg.-General—Colonel<br>One Purveyor-General—Colonel<br>One Inspector-General—Colonel<br>Twelve Staff Surgeons—Lieut.-Colonels | of Cavalry: |

One Surgeon (Major); one Assistant-Surgeon (Captain), and one Assistant-Surgeon (1st Lieut.) for each regiment of Artillery, Infantry, and Cavalry, for the Ordnance and for the Engineer Corps:—

2. For the Volunteer Army whenever called into service:—

One Assist.-Surg.-General, one Purveyor-General, and one Inspector-General: Staff Surgeons as many as are necessary for the command of the General Hospitals, with the same rank as the same officers in the regular service:—

One Surgeon (Major), one Assistant-Surgeon (Captain), and one Assistant-Surgeon (1st Lieut.) for each Regiment of the Line actually in service.

Candidates for the appointment of Assistant-Surgeon shall be examined by a board of not less than three Medical Officers of their own branch of the service.

A second examination by a similar board shall be required for promotion to the rank of Major.

General Hospitals and their Guards shall be under the command of the medical officer in charge, subordinate only to the commander of the Department or Army in which they are located.

A Hospital Corps, consisting of companies of sixty men each, shall be organized as companies of Infantry for duty as nurses, cooks, and guards to General Hospitals.

Promotions in the Medical Department of the Regular Army and volunteers shall be made in accordance with the principles that govern in other departments of the respective forces.

The Medical Director of an army in the field or a Geographical Military Department shall have the local rank of Colonel of Cavalry.

Medical Purveyors of principal Depôts (Surgeons in the regular Army of Volunteers) shall have the local rank of Colonel of Cavalry.

Physicians employed by contract shall have the local rank of 1st Lieutenant at the Hospitals or Posts where employed.

All acts or parts of acts inconsistent with this act are to be repealed.

We have the honor to be, very respectfully,

Your obedient servants,

(Signed) CHAS. S. TRIPLER, M.D.,
CHRIS. C. COX, M.D.,
FRANK H. HAMILTON, M.D.,
D. L. MCGUGEN, M.D.

In preparing this memorial and draft of a law, your Committee found it difficult to reconcile conflicting views and interests, the natural result of the distinct organizations, regular and volunteer, then in the service. They did not think that a law applicable to the then existing state of the department only, would fulfil the expectations of the Association. What was wanted was a plan for a permanent and not a merely temporary organization; a plan capable of being contracted to the limits of a peace establishment, still preserving its integrity, and, at the same time, of being instantaneously expanded sufficiently to meet the requirements of an army large enough to carry on any war in which the United States may hereafter be engaged.

It is evident that in time of peace, none other than regular troops can compose the army of the United States—it is equally evident that in time of war the greater portion of the troops in service must be composed of volunteers. If a plan had been proposed to merge the medical departments of the two descriptions of troops into one, and if such plan could be successful, it is plain that upon the reduction of the army all would become regular again, and upon the next occasion the distinction would be renewed and its conflicting interests and antagonisms, if they exist, must inevitably be revived.

The great objects of your Committee have been, first, to provide a medical staff for the land forces of the United States adequate to any emergency that may arise; and, second, to clothe the officers of that staff with a military rank that would forever settle the question of their subordination to officers of inferior rank in the other branches of the service.

Previously to the breaking out of the rebellion, the largest armies we have ever set on foot were insignificant in comparison with the enormous hosts that have been kept in the field for the last four years. For the last fifty years, with the exception of the Mexican War, the United States may be considered to have enjoyed uninterrupted peace. Our small force was distributed in scattered posts over the vast surface of the country, in detachments so small as to require little of a medical department beyond the mere prescribing for a dozen men daily at a post hospital. The military mind of the country could perceive no necessity for any military authority being conferred upon a medical officer, and this mind had indolently and thoughtlessly drifted into the idea that such an authority was preposterous and absurd. The school of the last four years has, however, done much toward dissipating this delusion. The wise and thoughtful, particularly in the higher grades, no longer cling to this mouldy fossil, and the present Secretary of War, by conferring brevets upon several medical officers, both regular and volunteer, and the Congress of the United States by enacting a law conferring the unlimited rank of colonel and lieutenant-colonel upon medical directors, has settled the principle that the medical staff is a military branch of the service, equally with the quartermaster's and subsistence departments.

The medical profession owes much to the Hon. E. M. Stanton, Secretary of War, for the several orders he has issued and enforced, conferring the independent command of the general hospitals upon the surgeon in charge, and placing under their command the officers and men constituting the Second Battalion of the Veteran Reserve Corps, thus removing a military disability from medical men, as fatal to their efficiency as it was insulting to their self-respect.

But the laws in reference to the present organization of the medical department of the army, with the exception of that in relation to medical directors, expire by their own limitation with the present war; when the country is declared pacified, things revert to their *status ante bellum*, and without further legislation, the people and armies of the United States are doomed again to encounter the confusion and disappointments necessarily consequnet upon the entirely inadequate organization of the medical department for a state of war.

By attending to this matter now, and by making suitable provisions for a medical department, susceptible of expansion to meet every emergency, this confusion and disappointment may be avoided, our troops always provided with a competent hospital establishment, the mode of appointment of medical officers fixed by law, and not left to the caprices of politicians and the chances of a ballot, and thousands of lives and millions of money saved to the nation.

By leaving the question of organization unsettled during the approaching interval of peace, which God grant may be a long one, we are certainly doomed to incur, upon the breaking out of another war, the same evils that have marked the inception and progress of this, and we shall require a repetition of the same severe lesson of suffering, to educate once more the military mind of the country up to a point that will induce it to yield a tardy and reluctant assent to the now patent fact—that the medical department is as essential to the organization of an army as the engineers, and that an efficient medical department cannot exist without a recognized military position. With these views your Committee would be insensible to the teachings of the past and to your just expectations, if they should fail to urge upon the members of the American Medical Association their earnest recommendation that, as an Association, they shall reaffirm their convictions of the necessity for immediate legislation in reference to the military rank of the medical department, and as individuals each shall pledge himself to use all honorable influences with the Representatives of their several Congressional districts, and the United States Senators from their States, to induce them, at the next session of Congress, to enact the law sketched out in the draft of your Committee, or some other law based upon the same fundamental principle.

All of which is respectfully submitted.

Charles S. Tripler, M.D.,
Chris. C. Cox, M.D.

